# Suggestions of a Method of Changing Bodily Form by the Administration of Certain Food

**Published:** 1891-02

**Authors:** 


### Suggestions of a Method of Changing Bodily Form by the Administration of Certain Foods.

That the nature and quantity of food taken by an animal modifies its economy in many directions is well known. That it is capable of inducing changes hitherto possible only by cross breeding is probably new to most. Prof. E. Fischer (*British Medical Journal*), the chemist who has devised the synthetic production of grape sugar, fruit sugar, and a whole series of new sugars, touches upon some of the problems of mutual interest to the chemist and physiologist. He says:

Next to the albumens the hydro-carbons form the chief food of the animal kingdom. Many valuable observations have been made concerning the processes they undergo in the animal body. What would be the result if artificial sugars were substituted for the natural hydro-carbons? Mannose, so closely related to grape sugar, and so easily fermented by yeast, might very probably form a good food stuff, even for the more highly organized animal body; and yet the slight change in substance might cause corresponding changes in the vital processes. If mannose be taken as food will the liver produce a new glycogen, and the mammalian gland a substitute for milk sugar; and will this sugar be oxydized in the body of the diabetic? The changes in the animal organism could not but be still more decided, if one could succeed in feeding the animal body with a pentose or a heptose, or more easily fermentable nonose. One would then probably find that blood and tissues would modify their functions, that the pig and goose would produce a changed fat; the bee a changed wax. Indeed, the experiment might be carried still farther. The assimilating plant prepares from sugar not only the more complicated hydro-carbons and the fats, but also with the help of inorganic nitrogenous compounds, the albuminoids. Certain classes of bacilli have the same power. Now, if it were possible to feed the assimilating plant of these bacilli with a differently constituted sugar they might possibly be forced to form a changed albumen! May we not then expect that changed building material will lead to changed architecture?

We should thus gain a chemical influence on the foundation of the organism which would necessarily lead to the most extraordinary phenomena, to changes in form far exceeding all that has been reached by cross breeding, etc. Since the fundamental experiments of Wohler and Frerichs, physiological chemists have incorporated hundreds of organic substances with the animal body, seeking the products in the urine; but they almost exclusively have used substances having no likeness to natural food stuffs. The use of the new series of sugars offers a wide field of action to the physiologist, and may be attended with results far more extraordinary. Biology here stands before a problem which could not have been until chemistry had prepared material for the experiment.

CAMPHORATED NAPHTHOL.—This mixture is composed of one part of naphthol and two parts of camphor, triturated together *dry*. Delsesquells discovered that naphthol liquefies in camphor, and M. Bouchard has shown the considerable antiseptic power of naphthol, and its great advantage in being non-toxic. He advises the use of camphorated naphthol as a topical antiseptic, having used it in many cases of excoriations, wounds, and ulcerations, and in diphtheria, as an application to the throat.—*Jour. Amer. Med. Asso.*

EMBRYOLOGY.—At the Leeds meeting of the British Association for the Advancement of Science, Professor Milnes Marshall, in his presidential address to the Biological Section, said that embryology had thriven mightily of late years; but the watching of all the processes of development was of trifling account compared with the great generalization, which showed that the development of animals had a far higher meaning. The phases through which an animal passed in its own development were no accidental freaks, but represented more or less closely, in more or less modified manner, the successive ancestral stages through which the present condition had been acquired. Evolution told us that each animal had had a pedigree in the past embryology revealed to us this ancestry, because every animal in its own development repeated this history. Such was the recapitulation theory. This had been hinted at by Agassiz, suggested more directly by von Baer, but first clearly enunciated by Fritz Muller; it has since been elaborated by many, notably by Balfour and by Ernest Haeckel. "Natural selection" explained the preservation of useful variations, but it did not account for the formation and preservation of useless organs. "Recapitulation" solved the problem at once by showing that these organs, though now useless, must have been of functional value to the ancestors of their present possessors, and that their appearance in existing forms was due to repetition of ancestral characters. Man himself presented many such features. The existence in the human adult of ear muscles which he did not use, of gill clefts in the embryo that never possessed functional importance, furnished suggestions of past history identical in the lesson they gave with that which the linguist found in the retention of a silent consonant in some words. The principle of degeneration, also, should not be overlooked. Professor Marshall emphasized the importance of Kleinenberg's theory as to the develpment of new organs, but said it was yet too early to realize the full significance of it. Embryology, though, could not provide an immediate or complete answer to the great riddle of life; it was a means; not an end.—*Medical Record.*

